# Circular RNA circNUP214 serves as a microRNA-31 sponge to promote the progression of myasthenia gravis through NFAT5

**DOI:** 10.3389/fneur.2026.1807844

**Published:** 2026-07-09

**Authors:** Fanfan Xu, Xiaotong Kong, Shanshan Peng, Lifang Li, Wenqi Tian, Hanlu Cai, Yichen Li, Ying Li, Jingyan Niu, Nan Zhang, Huixue Zhang, Lihua Wang

**Affiliations:** Department of Neurology, The Second Affiliated Hospital of Harbin Medical University, Harbin, China

**Keywords:** circNUP214, circular RNA, ceRNA, myasthenia gravis, NFAT5

## Abstract

Myasthenia gravis (MG) is an autoimmune disease driven by autoantibodies targeting the neuromuscular junction, leading to muscle weakness. Although emerging evidence implicates circular RNAs (circRNAs) in MG, their specific regulatory mechanisms remain largely unknown. In particular, the role of circNUP214 in MG has not been characterized. CircNUP214 expression in PBMCs and CD4^+^ T cells from MG patients and healthy controls was determined by qRT-PCR. Its circular structure and cytoplasmic localization were verified. Furthermore, the interaction between circNUP214 and miR-31 was confirmed, and the regulatory effect of circNUP214 on CD4^+^ T cell proliferation was evaluated. In 31 pure AChR antibody-positive MG patients, circNUP214 expression was significantly elevated in PBMCs compared with healthy controls (HC). Elevated circNUP214 expression was also observed in CD4^+^ T cells. Functionally, circNUP214 promoted CD4^+^ T cell proliferation, while its knockdown suppressed this process. Mechanistically, circNUP214 directly bound miR-31 and upregulated NFAT5 via sponging miR-31. Rescue experiments in Jurkat cells further validated the circNUP214/miR-31/NFAT5 ceRNA regulatory axis. In conclusion, circNUP214 is highly expressed in PBMCs from 31 pure AChR antibody positive MG patients and is also elevated in CD4^+^ T cells. It upregulates NFAT5 expression by sponging miR-31 to promote proliferation. These findings suggest that the circNUP214/miR-31/NFAT5 axis may contribute to aberrant CD4^+^ T cell proliferation in MG.

## Introduction

Myasthenia gravis (MG) is an autoimmune disorder characterized by weakness in the ocular, respiratory, limb, and bulbar muscles (1). Several autoantibodies, including those against the acetylcholine receptor (AChR), muscle-specific kinase (MuSK), and lipoprotein-related protein 4 (LRP4), have been implicated in the pathogenesis of MG (2). While autoantibodies against postsynaptic proteins are central to disease pathology, dysregulated T-cell responses play a pivotal role in initiating and perpetuating the autoimmune cascade (3). Specifically, CD4^+^ T cells are over-activated in MG patients (3); moreover, CD4^+^ T helper (Th) cells, particularly Th17 and follicular helper T (Tfh) subsets, drive pathogenic inflammation by promoting B-cell differentiation into autoantibody-producing plasma cells (4, 5). Consequently, the aberrant activation and proliferation of CD4^+^ T cell are regarded as central events in MG pathogenesis.

Circular RNAs (circRNAs) are a family of unique RNA molecules generated by back-splicing of pre-mRNAs, resulting in closed loop structures (6). Once considered splicing artifacts, circRNAs are now recognized as key regulators of gene expression, acting through multiple mechanisms including modulation of parental gene transcription, miRNA sponging, and regulation of RNA-binding proteins (7). Notably, circRNAs can function as competitive endogenous RNAs (ceRNAs), a mechanism through which they sequester miRNAs and relieve the post-transcriptional suppression of downstream targets, including transcription factors and immune regulators (8). Emerging evidence indicates that circRNAs are critical regulators in autoimmune diseases (9). For instance, in systemic lupus erythematosus (SLE), hsa_circ_0012919 has been shown to modulate MDA5 expression in CD4^+^ T cells by targeting miR-125a-3p, revealing the intrinsic regulatory role of circRNAs in T-cell biology (10). Concurrently, recent studies have highlighted the role of nuclear factor of activated T-cells 5 (NFAT5) as a key modulator of immune homeostasis, playing an essential role in restraining pro-inflammatory T-cell responses (11, 12). Its specific deletion in T cells leads to enhanced IFN-*γ*/IL-17 production and exacerbates autoimmune disease, further solidifying its critical function in maintaining immune tolerance (13).

In MG, a growing number of circRNAs have been reported to be dysregulated (14, 15); however, the molecular mechanisms by which circRNAs contribute to CD4^+^ T cell dysfunction remain poorly understood. In particular, little is known about circRNA-mediated regulation of NFAT5 in T cells. Therefore, this study aimed to explore the functional role of circNUP214 in MG and clarify its regulatory mechanism on CD4^+^ T cell proliferation via the miR-31/NFAT5 ceRNA axis.

## Subjects and methods

### Subjects and samples

A total of 40 patients with MG and 40 age- and sex-matched healthy controls (HC) were recruited from the Second Affiliated Hospital of Harbin Medical University. All healthy individuals had no history of neurological, autoimmune, inflammatory, cardiovascular or other systemic diseases. MG diagnosis was established strictly according to the 2020 Chinese Guidelines for the Diagnosis and Treatment of Myasthenia Gravis. Diagnostic criteria included typical fluctuating muscle weakness combined with at least one supportive evidence: pharmacological response, electrophysiological alteration, or positive serum autoantibodies. A definitive MG diagnosis was confirmed when typical clinical manifestations were validated by pharmacological and/or neuroelectrophysiological examinations. Exclusion criteria were as follows: (1) Incomplete clinical data; (2) Concomitant autoimmune diseases listed by the Global Autoimmune Institute (GAI); (3) Severe comorbidities, including heart disease, hepatic or renal insufficiency, or malignant neoplasms (with the exception of thymoma); (4) Receipt of immunotherapy or high-dose steroid pulse therapy within the past month. Peripheral blood samples were collected from all participants. The study was approved by the Ethics Committee of the Second Affiliated Hospital of Harbin Medical University, and all participants provided written informed consent.

### RT-qPCR

Human peripheral blood mononuclear cells (PBMCs) were isolated by density-gradient centrifugation over Ficoll–Hypaque solution (TBD, Tianjin, China) and stored at −80 °C. Total RNA was extracted from PBMCs using TRIzol® Reagent (Sigma Life Science, Darmstadt, Germany). RNA concentration was measured with a NanoDrop ND-1000 (Thermo Fisher Scientific). Total RNA was reverse-transcribed into cDNA with the PrimeScript RT Reagent Kit (Roche, Basel, Switzerland) following the manufacturer’s protocols. Quantitative real-time PCR (qRT-PCR) was carried out using FastStart Universal SYBR Green Master Mix (Roche, Basel, Switzerland) to detect the expression of mRNAs and circRNAs. Relative expression levels were calculated by the 2^−ΔΔCt^ method. The primer sequences used are listed in Table 1.

**Table 1:** Primers used for qRT-PCR analysis.

Target	Sequence (5′–3′)	Use/Direction
GAPDH	GAAGGTCGGTGAACGGAT	Forward (5′–3′)
	CCCATTTGATGCGGGAT	Reverse (5′–3′)
NFAT5	GTCACCGACAGCAAGGCTAT	Forward (5′–3′)
	AAGACTGTGTGCCTCTTCGG	Reverse (5′–3′)
circNUP214	CCTCTCTCAGCACCACCTAG	Forward (5′–3′)
	CAGCACTAAATCCCTGGGG	Reverse (5′–3′)
NUP214	CTTCCCAGAGCAGCATTCAC	Forward (5′–3′)
	TTGGGCAAGGATTTGGTGTG	Reverse (5′–3′)
miR-31	GTCGTATCCAGTGCAGGGTCCGAGGTATTCGCACTGGATACGACAGCTAT	Stem–loop RT primer
miR-31	AGGCAAGATGCTGGCATAG	Forward primer
miR-31	CAGTGCGTGTCGTGGAGT	Universal reverse primer
U6	CTCGCTTCGGCAGCACA	Forward (5′–3′)
U6	AACGCTTCACGAATTTGCGT	Reverse (5′–3′)

**Table 2:** Oligonucleotides for functional assays.

Name	Sequence (5′–3′)	Strand/Modification	Use
si-circNUP214#1	UCUCCCCAGGGAUUUAGUGCU	Sense	Knockdown
	AGCACUAAAUCCCUGGGGAGA	Antisense	
si-circNUP214#2	GCCAGGCUCUCCCCAGGGAUU	Sense	Knockdown
	AAUCCCUGGGGAGAGCCUGGC	Antisense	
si-circNUP214#3	CUCUCCCCAGGGAUUUAGUGC	Sense	Knockdown
	GCACUAAAUCCCUGGGGAGAG	Antisense	
si-circNUP214-NC	UUCUCCGAACGUGUCACGUTT	Sense	Negative control (siRNA)
	ACGUGACACGUUCGGAGAATT	Antisense	
miR-31 mimic	AGGCAAGAUGCUGGCAUAGCU	Sense	Gain of function (mimic)
	AGCUAUGCCAGCAUCUUGCCU	Antisense	
mimic-NC	UUCUCCGAACGUGUCACGUTT	Sense	Negative control (mimic)
	ACGUGACACGUUCGGAGAATT	Antisense	
miR-31 inhibitor	AGCUAUGCCAGCAUCUUGCCU	2′-O-methyl (2′-OMe) modified	Loss of function (inhibitor)
inhibitor-NC	CAGUACUUUUGUGUAGUACAA	2′-O-methyl (2′-OMe) modified	Negative control (inhibitor)

### Ribonuclease R

Total RNA (2 μg) extracted from Jurkat cells was treated with RNase R (Geneseed, Guangzhou, China) at 2 U/μg. The mixture was incubated at 37 °C for 10 min, followed by inactivation at 70 °C for 15 min. After RNase R digestion, the expression levels of circNUP214 and linear NUP214 mRNA were detected via qRT-PCR.

### Nuclear and cytoplasmic extraction

Cell lysates and nuclear/cytoplasmic fractions were prepared using the Nuclear and Cytoplasmic Protein Extraction Kit (Beyotime Biotechnology, Nanjing, China) in accordance with the manufacturer’s instructions. Freshly isolated cells were washed with ice-cold PBS and lysed in 200 μL of pre-chilled Cell Fractionation Buffer on ice for 10 min. After centrifugation at 500×g for 5 min at 4 °C, the cytoplasmic supernatant and nuclear pellet were collected separately.

### Cell culture and transfection

Jurkat cells (a human CD4^+^ T-cell line) were obtained from BeNa Culture Collection (Henan, China). Jurkat cells were maintained in RPMI 1640 (Gibco, Grand Island, NY, United States) medium. Both media were supplemented with 10% fetal bovine serum (Excell Bio, Suzhou, China) and 1% penicillin–streptomycin (Beyotime Biotechnology, Nanjing, China).

Small interfering RNAs (siRNAs) targeting circNUP214 were synthesized by GenePharma (Shanghai, China). The circNUP214 overexpression plasmid, miR-31 mimic, miR-31 inhibitor, and their respective negative controls (NCs) were all purchased from the same manufacturer. A non-specific scramble siRNA was used as an additional negative control in all experiments. All sequences are listed in Table 2. Exponentially growing Jurkat cells were harvested by centrifugation at 1000 rpm for 5 min, then seeded into 6-well plates at a density of 1 × 10^5^cells/well. Cell transfection was performed using Lipofectamine™ 3000 (Invitrogen, Carlsbad, CA, United States) following the manufacturer’s protocols. Transfected cells were cultured for 48 h before subsequent analysis. Transfection efficiency was verified by qRT-PCR.

### Luciferase reporter assay

The CircInteractome database was used to predict potential miR-31 binding sites on the circNUP214 sequence. Wild-type (WT) and mutant (MUT) circNUP214 fragments harboring the predicted and mutated binding sites were synthesized and subcloned into the psiCHECK-2 vector. For the luciferase reporter assay, these circNUP214 WT and MUT constructs were co-transfected into Jurkat cells with miR-31 mimic or negative control (miR-NC) using Lipofectamine® 3,000 Reagent. After 48 h of incubation, the luciferase activities of firefly and Renilla in harvested cells were measured with a dual-luciferase reporter assay system (Promega, Madison, WI, United States) following the manufacturer’s instructions.

### Flow cytometry and cell sorting

CD4^+^ T cells were isolated from PBMCs via fluorescence-activated cell sorting (FACS). Briefly, PBMCs were stained with fluorophore-conjugated anti-human CD3 and CD4 antibodies (eBioscience) for 30 min on ice. Cell sorting was conducted using a Beckman Coulter MoFlo Astrios cell sorter. The live, CD3^+^CD4^+^ T-cell population was gated and collected. The sorted cells, with a confirmed purity of >98%, were collected directly into lysis buffer for subsequent RNA extraction.

### Western blotting analysis

Total protein was extracted from cells using RIPA lysis buffer, and protein concentration was quantified with a BCA Assay Kit (Beyotime Biotechnology, Nanjing, China). Protein samples were separated by SDS-PAGE and transferred onto PVDF membranes. Membranes were blocked with 5% non-fat milk for 2 h at room temperature, then incubated with specific primary antibodies overnight at 4 °C. After thorough washing with TBST, membranes were incubated with secondary antibodies for 1 h. Immunoreactive bands were visualized using an ECL kit (GE Healthcare). Primary antibodies used in this study included NFAT5 (Immunoway, YT5853) and *β*-actin (Abclonal, AC026).

### Cell proliferation

Cell proliferation was assessed using the Cell Counting Kit-8 (CCK-8, Seven, Beijing, China) and 5-ethynyl-2′-deoxyuridine (EdU) Kit (Beyotime, Shanghai, China). For the CCK-8 assay, Jurkat cells were seeded into 96-well plates (3,000 cells/well) 24 h after transfection and cultured at 37 °C with 5% CO₂. At the indicated time points, 10 μL of CCK-8 reagent was added to each well and incubated for 2 h. Absorbance (optical density, OD) was measured at 450 nm using a microplate reader (BioTek, Vermont, United States).

EdU incorporation was assessed using an EdU Kit according to the manufacturer’s protocol. Cells were incubated with 10 μM EdU for 2 h, fixed with 4% paraformaldehyde for 15 min, and permeabilized with 0.3% Triton X-100 for 10 min. After incubation with Click Additive Solution (Azide 594) for 30 min, nuclei were counterstained with Hoechst 33342 for 10 min. Images were acquired using a fluorescence microscope (Nikon, Japan).

### Statistical analysis

All samples and experiments were assigned using randomization to minimize selection bias. Blinding was implemented during sample testing and data analysis, with researchers blinded to group information.

All statistical analyses and graphing were performed using GraphPad Prism 9.0 software. Data normality was first evaluated. For two-group comparisons, an unpaired Student’s *t*-test (normally distributed data) or Mann–Whitney *U* test (for non-normally distributed data) was employed. One-way ANOVA followed by Tukey’s *post hoc* test was applied for multiple-group comparisons. Correlations were assessed using Pearson’s (normally distributed data) or Spearman’s (non-normally distributed data) coefficients. Statistical significance was defined as *p* < 0.05 (*p < 0.05, **p < 0.01, ***p < 0.001, ****p < 0.0001).

## Results

### Characterization of circNUP214 function in Jurkat cells

**Figure 1:**
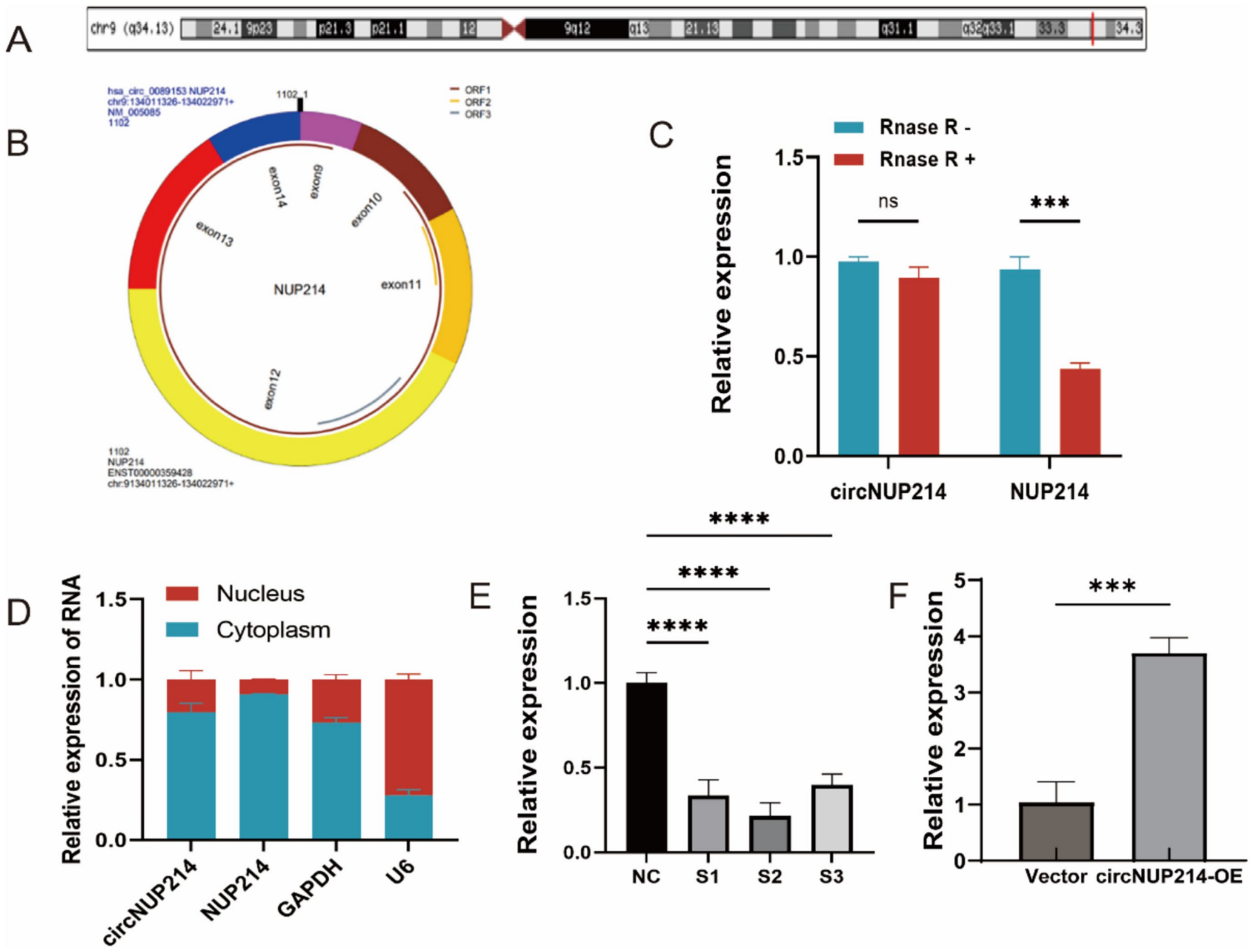
Characterization of circNUP214 in Jurkat cells. **(A,B)** Genomic position of circNUP214 on the human chromosome. **(C)** Relative expression of circNUP214 and linear NUP214 mRNA in Jurkat cells with or without RNase R treatment, measured by qRT-PCR. **(D)** Cytoplasmic and nuclear fractionation showing circNUP214 is predominantly localized in the cytoplasm of Jurkat cells. **(E,F)** Knockdown and overexpression efficiency of circNUP214 in Jurkat cells, measured by qRT-PCR. Data are presented as mean ± SD across 3 independent biological experiments, each with 3 technical replicates per group (***p < 0.001, ****p < 0.0001).

The circNUP214 transcript (chr9:134011326–134022971) represents an exonic circular RNA that maps to chromosome 9q34 (Figures 1A,B). The transcript hsa_circ_0089153 was designated as circNUP214 based on its parental gene. To evaluate its transcript stability, we assessed the resistance of circNUP214 and its linear counterpart, NUP214 mRNA, to RNase R digestion (Figure 1C). Its resistance to RNase R treatment confirmed the circular nature of this transcript. Subsequent nuclear and cytoplasmic fractionation revealed that circNUP214 was predominantly localized in the cytoplasm (Figure 1D). Together, these findings demonstrate that circNUP214 is a stable circular RNA enriched in the cytoplasm. Among the three designed siRNAs targeting circNUP214, the S2 construct exhibited the highest knockdown efficiency; therefore, it was selected for subsequent experiments (Figure 1E). Furthermore, qRT–PCR analysis confirmed that circNUP214 overexpression significantly elevated its transcript levels (Figure 1F).

### CircNUP214 is upregulated and clinically correlated in MG patients

To examine circNUP214 expression profiles, peripheral blood samples were collected from MG patients and healthy controls (HC). The clinical characteristics of all participants are summarized in Table 3. In total, 40 MG patients and 40 HC were enrolled, with no significant differences in age or gender distribution between the two groups (*p* > 0.05). Among MG patients, 6 were classified as early-onset MG (EOMG) and 34 as late-onset MG (LOMG). According to the Myasthenia Gravis Foundation of America (MGFA) classification, 15 patients were Class I (ocular type), 23 were Class II, 1 was Class III, and 1 was Class IV; no Class V cases were included.

**Table 3:** Demographic and clinical characteristics of MG patients and healthy controls.

Characteristic	MG patients	Healthy controls	*p*-value
Number	40	40	—
Age, years, mean ± SD	60.00 ± 11.60	59.00 ± 8.65	0.6632
Sex, male, *n*	22	20	0.654
EOMG, *n*	6		
LOMG, *n*	34		
MGFA, *n*			
I	15		
II	23		
III	1		
IV	1		
V	0		
Antibody, *n*			
AChR	31		
MuSK	0		
AChR + Titin	3		
AChR + Titin + RyR	4		
Undetected	2		
Duration (years), *n*			
<1	22		
1–5	13		
>5	5		
Comorbidity, *n*			
Thyroid disease	5		
Abnormal thymus (thymic hyperplasia or thymoma)	8		
Treatment			
Only symptomatic drug treatment	12		
Immunosuppressive drug treatment	26		
Thymectomy	9		
IVIG/PLEX	15		

Symptomatic treatment refers to pyridostigmine. Immunosuppressive treatment includes glucocorticoids, azathioprine, mycophenolate mofetil, and tacrolimus. MG, myasthenia gravis; HC, healthy controls; SD, standard deviation; EOMG, early-onset myasthenia gravis (age ≤50 years); LOMG, late-onset myasthenia gravis (age >50 years); MGFA, Myasthenia Gravis Foundation of America classification; AChR, anti-acetylcholine receptor antibody; MuSK, anti-muscle-specific kinase antibody; RyR, anti-ryanodine receptor antibody; IVIG, intravenous immunoglobulin; PLEX, plasma exchange.

In terms of autoantibody profiles, 31 patients were anti-AChR positive, 3 were positive for both anti-AChR and anti-Titin, 4 were positive for anti-AChR, anti-Titin, and anti-RyR, and 2 were seronegative; no anti-MuSK-positive patients were enrolled. Regarding prior treatment history, 12 patients received only symptomatic therapy with pyridostigmine alone, while 26 were treated with immunosuppressive agents including glucocorticoids, azathioprine, mycophenolate mofetil, and tacrolimus. Nine patients underwent thymectomy, and 15 received intravenous immunoglobulin (IVIG) and/or plasma exchange (PE), with several patients receiving combined multiple immunotherapies.

To explore its potential clinical relevance, we further stratified circNUP214 expression according to MGFA classification and autoantibody subtype (Supplementary Table S1). No significant difference in circNUP214 expression was observed between ocular (Class I) and generalized (Class II–IV) MG subgroups (median: 1.31 vs. 1.35; *p* = 0.252). Likewise, expression levels were comparable between patients with isolated AChR positivity and those with combined autoantibody positivity (AChR plus Titin and/or RyR; median: 1.34 vs. 1.24; *p* = 0.619). All subsequent analyses were performed on the 31 AChR-antibody-positive MG patients unless otherwise specified. An expanded cohort of 38 patients (2 seronegative excluded) was also analyzed; the results are provided in Supplementary material 2.

**Figure 2:**
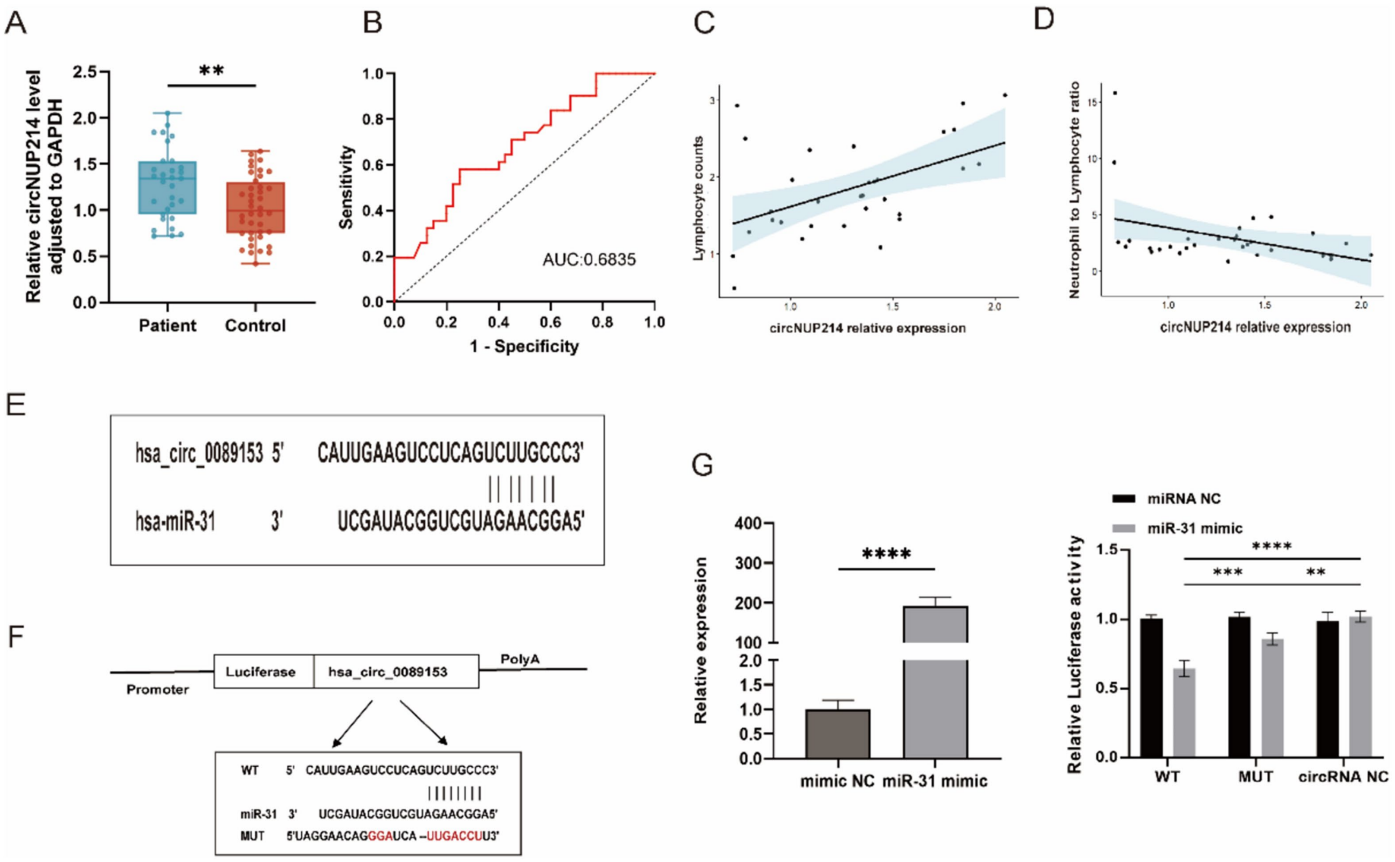
Upregulated circNUP214 in 31 pure AChR-antibody-positive MG patients and its direct interaction with miR-31. **(A)** The transcript levels of circNUP214 in PBMCs from MG patients (*n* = 31) and controls (*n* = 40) were determined by qRT-PCR. Each sample was run in triplicate (technical replicates). **(B)** ROC curve analysis showed that the circNUP214 expression levels have diagnostic value in MG patients. **(C)** Relationships between the expression levels of circNUP214 and the lymphocyte counts in peripheral blood from MG patients. Lymphocyte counts were measured by routine blood tests using an automated hematology analyzer, with units of ×10^9^/L. **(D)** Relationships between the expression levels of circNUP214 and the neutrophil to lymphocyte ratio in MG patients. **(E,F)** Binding sites between circNUP214 and miR-31 were predicted using the Circular RNA Interactome database. **(G)** Left: RT-qPCR validation of miR-31 expression in Jurkat cells after transfection with miR-31 mimic. Right: Relative luciferase activity of wild-type (WT) and mutant (MUT) circNUP214 reporters in Jurkat cells after co-transfection with miR-31 mimic or miRNA NC. For the left panel, an unpaired *t*-test was applied (***p < 0.001). For the right panel, we performed one-way ANOVA followed by Tukey’s multiple comparisons test. Pairwise comparisons were conducted within each treatment group, and all reported values are Tukey-adjusted *p*-values: **p < 0.01, ***p < 0.001, ****p < 0.0001. Data in **(A,G)** are presented as mean ± SD.

CircNUP214 expression levels were significantly upregulated in PBMCs from MG patients relative to healthy controls (Figure 2A; *p* < 0.001). Furthermore, a Receiver operating characteristic (ROC) curve analysis was further performed to evaluate the diagnostic potential of circNUP214 for MG in this cohort. The results indicated that circNUP214 could distinguish MG patients from HC. The AUC reached 0.6835 (95% confidence interval CI = 0.5599–0.8070, *p* < 0.01; Figure 2B). In addition, we investigated the association between circNUP214 expression levels and routine clinical blood parameters in patients. CircNUP214 expression was positively correlated with lymphocyte counts (*r* = 0.4935, *p* < 0.01; Figure 2C). Conversely, circNUP214 expression showed a negative correlation with the neutrophil-to-lymphocyte ratio (*r* = −0.3554; *p* < 0.05; Figure 2D). Together, these results suggest that aberrant circNUP214 expression may contributes to the clinical progression of MG.

### CircNUP214 is identified as a miR-31 target by in silico prediction and luciferase reporter assays in Jurkat T cells

In the cytoplasm, circRNAs frequently function as ceRNAs to sequester miRNAs. Using the CircInteractome database (16), we predicted that miR-31 could potentially bind to circNUP214 (Figures 2E,F). We subsequently confirmed the successful overexpression of miR-31 *in vitro*. Luciferase reporter minigenes carrying wild-type or mutant circNUP214 were generated to assess miR-31 binding to circNUP214 (Figure 2G). Dual-luciferase reporter assays showed that co-transfection of the WT circNUP214 reporter with miR-31 mimic significantly suppressed firefly luciferase activity (normalized to Renilla luciferase activity) relative to miR-NC. In contrast, the reduction in luciferase activity was attenuated in the MUT circNUP214 reporter group, while no change was observed in the circRNA-NC control group. These findings indicate that circNUP214 directly interacts with miR-31 via the predicted binding site.

### CircNUP214 functions as a miR-31 sponge to regulate NFAT5 expression in MG patients

**Figure 3:**
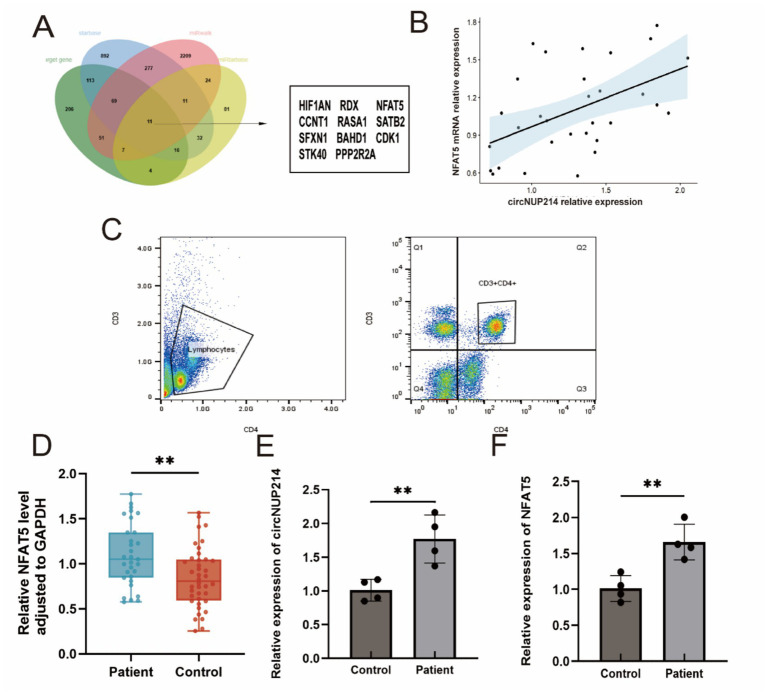
Correlation between NFAT5 expression and circNUP214 in MG patients. **(A)** Venn diagram showing potential target mRNAs of miR-31. **(B)** The correlation between circNUP214 expression and NFAT5 levels in 31 pure AChR-antibody-positive MG patients. **(C)** Representative gating strategy for CD4^+^ T cells (CD3^+^ CD4^+^). **(D)** The transcript levels of NFAT5 mRNA in PBMCs from 31 pure AChR-antibody-positive MG patients and controls (n = 40) were determined by qRT-PCR. Each sample was run in triplicate (technical replicates). **(E,F)** The expression levels of circNUP214 and NFAT5 were detected in CD4^+^ T cells from MG patients (*n* = 4) and healthy controls (*n* = 4) by qRT–PCR. Each individual subject served as one biological replicate, with three technical replicates per sample. Data are presented as mean ± SD. (**p < 0.01, ***p < 0.001, ****p < 0.0001).

To identify the downstream target of miR-31, we integrated prediction results from starBase (17), miRTarBase (18), TargetScan (19), and miRwalk (20). This integration yielded several candidate target genes, including HIF1AN, RDX, NFAT5, CCNT1, RASA1, SATB2, SFXN1, BAHD1, CDK1, STK40, and PPP2R2A, which were selected for further validation (Figure 3A). Among them, NFAT5 was prioritized because of its established role in T-cell activation and its involvement in multiple autoimmune diseases. To test whether circNUP214 could act as a ceRNA for NFAT5 via miR-31, no potential interaction between circNUP214 and NFAT5 was predicted by the online tool CSCD.^1^ We further analyzed and characterized shared miRNA response elements (MREs) of circNUP214 and NFAT5 mRNA using the miRanda algorithm with strict screening thresholds (score ≥140, ΔG ≤ −1 kcal/mol). Bioinformatic analysis verified that both transcripts harbor conserved and perfectly matched seed sequences for miR-31-5p (21). The predicted binding free energy was −15.65 kcal/mol for circNUP214 and −13.87 kcal/mol for NFAT5 mRNA, suggesting stable binding of both MREs (Supplementary material 1). The transcript levels of NFAT5 in MG patients were measured by qRT–PCR to analyze its expression correlation with circNUP214. NFAT5 expression was significantly elevated in PBMCs from MG patients (Figure 3D). Furthermore, circNUP214 levels showed a positive correlation with NFAT5 transcript levels (*r* = 0.4978, *p* < 0.01; Figure 3B). Subsequently, CD4^+^ T cells were isolated from peripheral blood mononuclear cells of MG patients and healthy controls. The expression levels of both circNUP214 and NFAT5 were detected by RT-qPCR. As shown in Figures 3E,F, both transcripts were significantly upregulated in CD4^+^ T cells from MG patients compared to healthy controls.

### CircNUP214 regulates NFAT5 through miR-31 sponging in Jurkat cells

**Figure 4:**
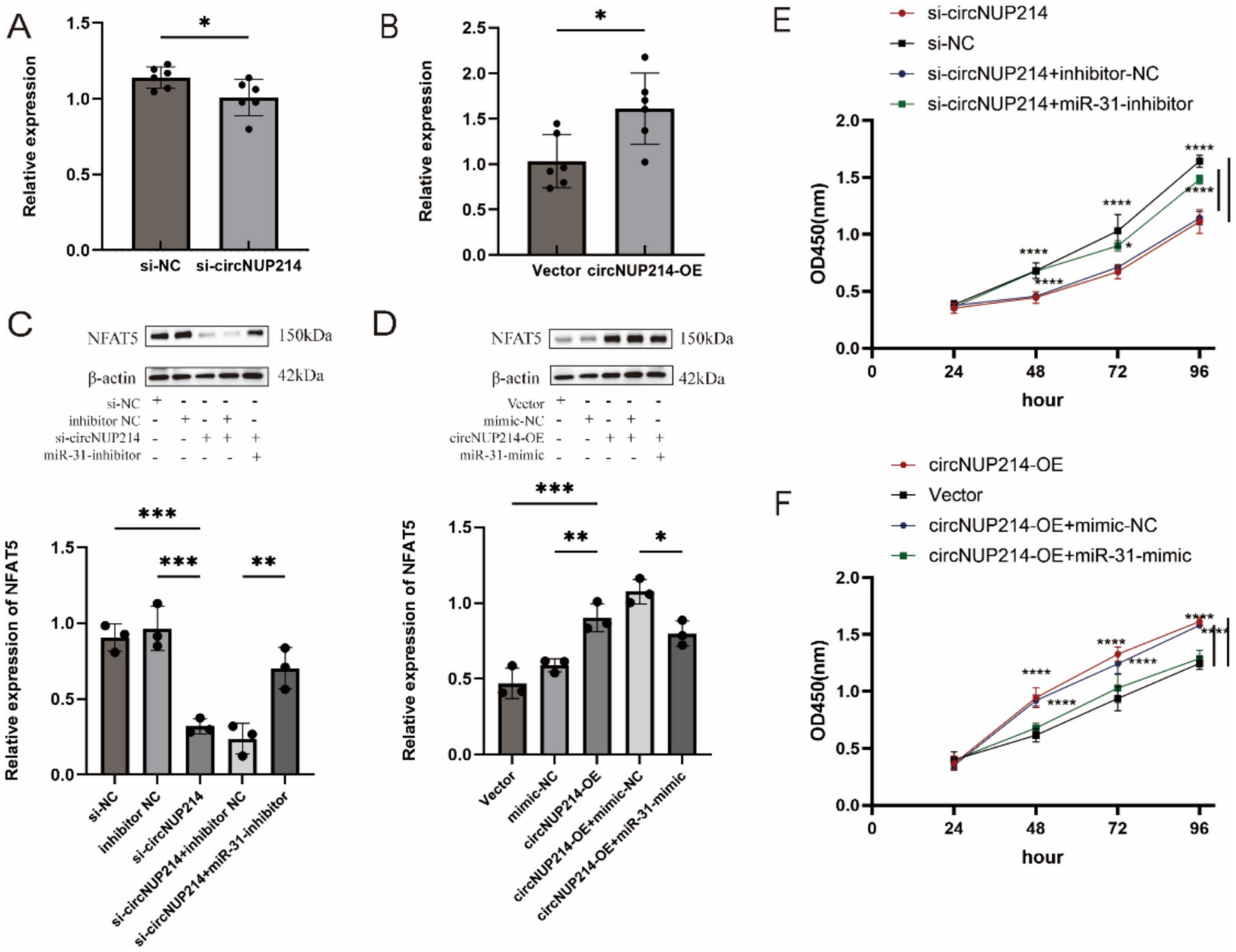
CircNUP214 modulates NFAT5 expression through miR-31. **(A,B)** qRT-PCR detection of NFAT5 mRNA expression in Jurkat cells transfected with NC, si-circNUP214 or circNUP214-OE (*n* = 6 biological replicates, each with three technical replicates). **(C,D)** Western blot analysis of NFAT5 protein levels in experimental (*n* = 3 biological replicates). **(E,F)** CCK-8 assays for the proliferation of Jurkat cells transfected with NC, si-circNUP214, circNUP214-OE, miR-31 inhibitor, or miR-31 mimic (*n* = 3 biological replicates, each with three technical replicates). Unpaired *t*-test was used for **(A,B)** (*p < 0.05). One-way ANOVA with Tukey’s test was used for **(C–F)**; adjusted *p*-values: *p < 0.05, **p < 0.01, ***p < 0.001, ****p < 0.0001. Data are mean ± SD.

To verify that circNUP214 regulates NFAT5 by sponging miR-31, we performed rescue experiments. qRT-PCR analysis revealed that knockdown of circNUP214 decreased NFAT5 mRNA levels (Figure 4A). Conversely, circNUP214 overexpression led to an increase in NFAT5 mRNA (Figure 4B). Notably, co-transfection with a miR-31 inhibitor reversed the knockdown-induced reduction of NFAT5 (Figure 4C). In contrast, the circNUP214 overexpression-induced upregulation of NFAT5 was mitigated by co-transfection with a miR-31 mimic (Figure 4D).

**Figure 5:**
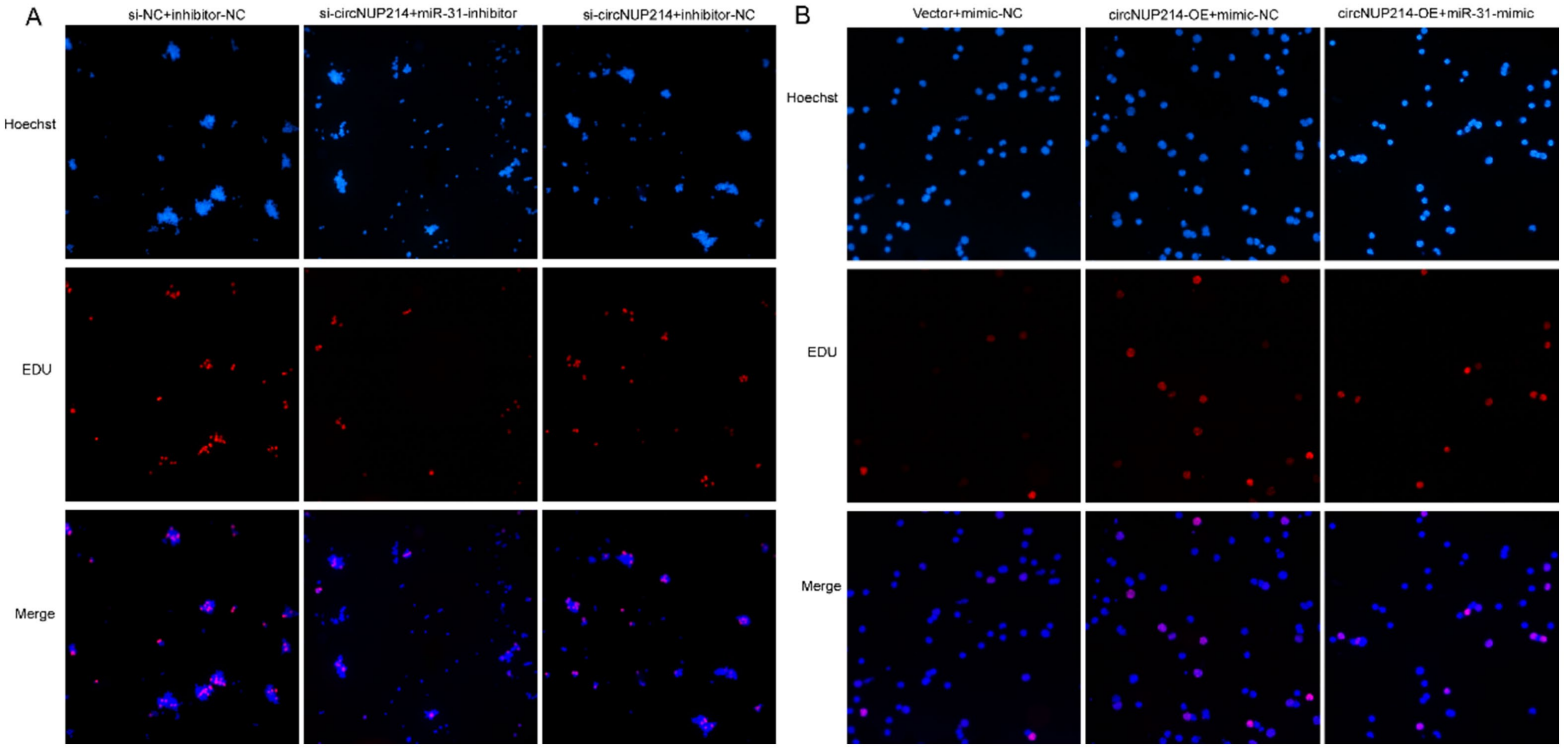
CircNUP214 promoted CD4+ T-cell proliferation. **(A,B)** EdU assay for Jurkat cells proliferation (*n* = 3 biological replicates; ≥3 randomly selected microscopic fields were analyzed per replicate).

To further validate the role of the circNUP214/miR-31/NFAT5 axis in the progression of MG, we investigated its impact on CD4^+^ T cell proliferation. The knockdown-induced reduction of NFAT5. In Jurkat T cells, knockdown of circNUP214 significantly suppressed proliferation; however, this suppression was rescued by co-inhibition of miR-31 (Figures 4E, 5A). Conversely, the enhanced proliferation induced by overexpression of circNUP214 was attenuated by a miR-31 mimic (Figures 4F, 5B). Collectively, the findings from the CCK-8 and EdU assays suggest that the circNUP214/miR-31 axis may play a role in regulating CD4^+^ T cell proliferation.

## Discussion

MG is a prototypical antibody-mediated autoimmune disease driven by autoantibodies targeting the neuromuscular junction. The production of these pathogenic autoantibodies is critically dependent on CD4^+^ T cell help (22). Consequently, the aberrant CD4^+^ T cell activation and proliferation constitute central events in breaking immune tolerance and initiating the autoimmune cascade in MG (23). In this context, our study provides preliminary evidence that circNUP214 may act as a miR-31 sponge to modulate NFAT5 expression, thereby potentially contributing to CD4^+^ T cell dysregulation in MG.

Previous circRNA studies in MG have mostly focused on expression profiling (24), while few have explored downstream molecular mechanisms or immune regulatory functions. Although circNUP214 has been reported to promote Th17-mediated pathogenesis in rheumatoid arthritis (25), its role in MG remains uncharacterized. Against this backdrop, our work presents several distinctive findings. First, we demonstrate the previously unreported upregulation of circNUP214 in both peripheral PBMCs and isolated CD4^+^ T cells from MG patients. Second, we experimentally characterize validated the circNUP214/miR-31/NFAT5 ceRNA axis in MG, a regulatory pathway not previously identified in this field of research. Third, the elevated circNUP214 expression and its trending association with generalized MG and multi-antibody-positive subgroups reveal a clinical phenotypic association that has not been documented before.

Numerous circRNAs exert biological functions via a typical ceRNA mechanism, whereby they act as molecular sponges to sequester miRNAs and relieve the post-transcriptional repression of miRNA target genes (26). The classic ceRNA regulatory paradigm was first established by the landmark finding that CDR1as can bind and sponge miR-7 to modulate downstream gene expression (27). Potential binding sites between circNUP214 and miR-31 were predicted using the Circular RNA Interactome database. Bioinformatic algorithms further identified several potential targets of miR-31, among which NFAT5 was prioritized for several functional reasons. NFAT5 has been shown to promote inflammatory responses in different immune cells by enhancing TLR-induced transcription of genes such as Nos2, Tnf, Il-6, and Il-1 (28). Functionally, NFAT5 acts as a downstream effector of the Neat1/miR-128-3p axis to drive antigen-specific Th17 responses (29). The pathogenic role of NFAT5 extends to several autoimmune diseases. In rheumatoid arthritis, NFAT5 facilitates dendritic cell maturation via the p38 MAPK pathway, leading to Th1/Th17 differentiation (30). In type 1 diabetes, the miR-181a/NFAT5 axis restrains the generation of FOXP3^+^ regulatory T cells (Tregs) and disrupts immune tolerance (31). In multiple sclerosis, NFAT5 haploinsufficiency reduces EAE severity and increases Treg frequency, suggesting that NFAT5 suppresses Treg cells to exacerbate neuroinflammation (32). Direct evidence linking NFAT5 to pathogenic Th17 cells comes from Kleinewietfeld et al. (33): high-salt conditions activate NFAT5 via p38 MAPK, and NFAT5 knockdown abrogates the development of highly pathogenic Th17 cells that produce GM-CSF, TNF-*α*, and IL-2. Furthermore, direct evidence connects NFAT5 to MG: Xin et al. (34) identified NFAT5 as a miR-20b target in thymoma-associated MG. NFAT5 was inversely correlated with miR-20b and modulated T-cell proliferation. Xu et al. (35) showed that NFAT5 binds to the G0S2 promoter and regulates lymphocyte balance in AChR-positive MG. Given its well-documented roles in inflammation and autoimmune disorders, we selected NFAT5 for further functional validation. Our follow-up assays confirmed that miR-31 directly binds to the 3′-UTR of NFAT5 and downregulated its expression (36).

Consistent with these bioinformatic predictions, our luciferase reporter assay experimentally verified the direct interaction between circNUP214 and miR-31. Furthermore, functional rescue experiments in Jurkat cells showed that miR-31 overexpression reversed the upregulation of NFAT5 induced by circNUP214 overexpression. In contrast, miR-31 knockdown further increased NFAT5 expression in circNUP214-overexpressed Jurkat cells. Collectively, these results demonstrate that circNUP214 upregulates NFAT5 expression by competitively sponging miR-31.

The enhanced CD4^+^ T cell proliferation driven by the circNUP214/miR-31/NFAT5 axis may contribute to MG pathogenesis through multiple pathways. First, hyperactivated CD4^+^ T cells, particularly Tfh cells, provide excessive B-cell help within germinal centers, promoting affinity maturation and the production of high-affinity anti-AChR autoantibodies. Elevated NFAT5 activity in CD4^+^ T cells may further enhance Tfh differentiation and function, thereby amplifying B-cell responses and aggravating autoantibody-mediated tissue damage at the neuromuscular junction. In addition, NFAT5 acts as a key regulator of pro-inflammatory cytokines including IFN-*γ*, IL-17, and TNF-*α*, which are well documented to exacerbate autoimmune inflammation in MG. Aberrant NFAT5 signaling in proliferating CD4^+^ T cells may skew immune homeostasis toward a pro-inflammatory state, promoting the recruitment and activation of additional immune cells.

Despite these insights, this study has several limitations. First, functional experiments were performed in Jurkat cells, which may not fully represent primary CD4^+^ T cells from MG patients. Second, we did not further explore the role of circNUP214 in specific CD4^+^ T cell subsets, such as Th1, Th17, Th2, or Treg cells. Third, the modest sample size reduced the statistical power for subgroup analyses. In addition, the validation of the circNUP214/miR-31/NFAT5 axis remains incomplete: direct binding between miR-31 and NFAT5 was not experimentally confirmed, and neither antagomir-based knockdown of miR-31 nor antago-circRNA-based knockdown of circNUP214 was performed due to technical and resource constraints. Future investigations will utilize primary CD4^+^ T cells to further explore the mechanism of circNUP214 in MG, and investigate its biological function in different CD4^+^ T cell subsets via flow sorting or single-cell sequencing. Furthermore, *in vivo* animal models will be employed to verify the circNUP214/miR-31/NFAT5 axis. Finally, larger patient cohorts will be required to validate the clinical relevance of these findings.

In conclusion, this study reveals that circNUP214 is highly expressed in PBMCs from 31 pure AChR antibody positive MG patients and is also elevated in CD4^+^ T cells. CircNUP214 acts as a ceRNA by sponging miR-31 to upregulate NFAT5, thereby promoting CD4^+^ T cell proliferation. The findings of this study may provide a valuable reference for understanding the role of circNUP214 in CD4^+^ T cell activation during the pathogenesis of MG.

## Data Availability

The original contributions presented in the study are included in the article/Supplementary material, further inquiries can be directed to the corresponding authors.

## Footnotes

1
https://geneyun.net/CSCD/
