# Supplementary material for: Circular RNA circNUP214 serves as a microRNA-31 sponge to promote the progression of myasthenia gravis through NFAT5
Source: Front Neurol. 2026 Jul 9;17:1807844. doi: 10.3389/fneur.2026.1807844 (PMC13391849; doi:10.3389/fneur.2026.1807844)
Supplement: Supplementary file 1 [file Data_sheet_1.docx]

Current Settings:

Query Filename: 163c55c2a0539240.miRNA.fa

Reference Filename: 163c55c2a0539240.utr.fa

Gap Open Penalty: -9.000000

Gap Extend Penalty: -4.000000

Score Threshold: 140.000000

Energy Threshold: -1.000000 kcal/mol

Scaling Parameter: 4.000000

=-=-=-=-=-=-=-=-=-=-=-=-=-=-=-=-=-=-=-=-=-=-=-=-=-=-=-=-=-=-=-=

Read Sequence: hsa-miR-31-5p (21 nt)

Read Sequence: hsa_circ_0089153|NM_005085|NUP214 (1102 nt)

=-=-=-=-=-=-=-=-=-=-=-=-=-=-=-=-=-=-=-=-=-=-=-=-=-=-=-=-=-=-=-=

Performing Scan: hsa-miR-31-5p vs hsa_circ_0089153|NM_005085|NUP214

=-=-=-=-=-=-=-=-=-=-=-=-=-=-=-=-=-=-=-=-=-=-=-=-=-=-=-=-=-=-=-=

Forward: Score: 148.000000 Q:2 to 18 R:976 to 997 Align Len (17) (70.59%) (76.47%)

Query: 3' ucgaUACGGUCGU-AGAACGGa 5'

| |:| || |||||||

Ref: 5' attgAAGTCCTCAGTCTTGCCc 3'

Energy: -15.650000 kCal/Mol

Scores for this hit:

>hsa-miR-31-5p hsa_circ_0089153|NM_005085|NUP214 148.00 -15.65 2 18 976 997 17 70.59% 76.47%

Score for this Scan:

Seq1, Seq2, Tot Score, Tot Energy, Max Score, Max Energy, Strand, Len1, Len2, Positions

>>hsa-miR-31-5p hsa_circ_0089153|NM_005085|NUP214 148.00 -15.65 148.00 -15.65 1 21 1102 976

Current Settings:

=-=-=-=-=-=-=-=-=-=-=-=-=-=-=-=-=-=-=-=-=-=-=-=-=-=-=-=-=-=-=-=

Query Filename: d2916330963fa820.miRNA.fa

Reference Filename: d2916330963fa820.utr.fa

Gap Open Penalty: -9.000000

Gap Extend Penalty: -4.000000

Score Threshold: 140.000000

Energy Threshold: -1.000000 kcal/mol

Scaling Parameter: 4.000000

=-=-=-=-=-=-=-=-=-=-=-=-=-=-=-=-=-=-=-=-=-=-=-=-=-=-=-=-=-=-=-=

Read Sequence: hsa-miR-31-5p (21 nt)

Read Sequence: Nfat5 (23 nt)

=-=-=-=-=-=-=-=-=-=-=-=-=-=-=-=-=-=-=-=-=-=-=-=-=-=-=-=-=-=-=-=

Performing Scan: hsa-miR-31-5p vs Nfat5

=-=-=-=-=-=-=-=-=-=-=-=-=-=-=-=-=-=-=-=-=-=-=-=-=-=-=-=-=-=-=-=

Forward: Score: 141.000000 Q:2 to 20 R:4 to 23 Align Len (18) (61.11%) (72.22%)

Query: 3' ucGAUACGGUCGUAGAACGGa 5'

:| || |: |||||||

Ref: 5' agUUUUGGGUGU-UCUUGCCa 3'

Energy: -13.870000 kCal/Mol

Scores for this hit:

>hsa-miR-31-5p Nfat5 141.00 -13.87 2 20 4 23 18 61.11% 72.22%

Score for this Scan:

Seq1, Seq2, Tot Score, Tot Energy, Max Score, Max Energy, Strand, Len1, Len2, Positions

>>hsa-miR-31-5p Nfat5 141.00 -13.87 141.00 -13.87 1 21 23 4
