# Supplementary material for: Circular RNA circNUP214 serves as a microRNA-31 sponge to promote the progression of myasthenia gravis through NFAT5
Source: Front Neurol. 2026 Jul 9;17:1807844. doi: 10.3389/fneur.2026.1807844 (PMC13391849; doi:10.3389/fneur.2026.1807844)
Supplement: Supplementary file 2 [file Data_sheet_2.docx]

**Supplementary material 2**


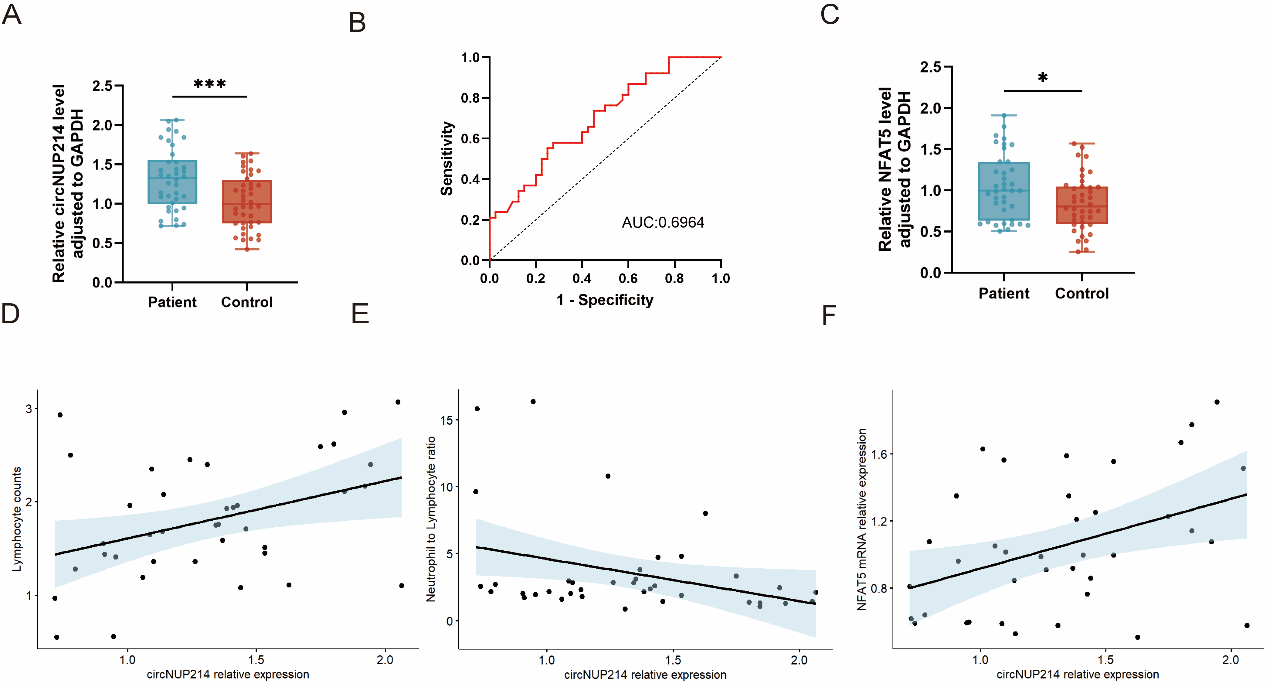


Figure S2 Expression and correlation analyses of circNUP214‑related signatures in the expanded MG cohort (n=38).

In the expanded cohort of 38 MG patients (2 seronegative excluded), circNUP214 expression in PBMCs was significantly increased compared with that in healthy controls (Fig. S2A; *p* < 0.001). ROC analysis yielded an AUC of 0.6964 (95% CI: 0.5815–0.8113, *p* < 0.01) for circNUP214‑based MG diagnosis (Fig. S2B). NFAT5 mRNA levels were also significantly upregulated in patient PBMCs (Fig. S2C; *p* < 0.05). CircNUP214 expression positively correlated with peripheral blood lymphocyte counts (*r* = 0.5005, *p* < 0.01; Fig. S2D) and NFAT5 transcript levels (*r* = 0.4122, *p* < 0.05; Fig. S2F), and negatively correlated with the neutrophil‑to‑lymphocyte ratio (*r* = −0.3221, *p* < 0.05; Fig. S2E).

**(A)** Relative circNUP214 expression in PBMCs from MG patients (n=38) and healthy controls (n=40) detected by qRT‑PCR. **(B)** ROC curve analysis for the diagnostic value of circNUP214 in MG. **(C)** Relative NFAT5 mRNA expression in PBMCs from MG patients (n=38) and healthy controls (n=40) detected by qRT‑PCR. **(D)** Correlation between circNUP214 expression and peripheral blood lymphocyte counts in MG patients. **(E)** Correlation between circNUP214 expression and neutrophil‑to‑lymphocyte ratio in MG patients. **(F)** Correlation between circNUP214 and NFAT5 expression in MG patients.
