# Supplementary material for: Circular RNA circNUP214 serves as a microRNA-31 sponge to promote the progression of myasthenia gravis through NFAT5
Source: Front Neurol. 2026 Jul 9;17:1807844. doi: 10.3389/fneur.2026.1807844 (PMC13391849; doi:10.3389/fneur.2026.1807844)

Schematic of circNUP214 overexpression construct. CircNUP214 sequence with flanking back-splicing elements was cloned into MCS of pcDNA3.1(+); arrows show transcription direction.


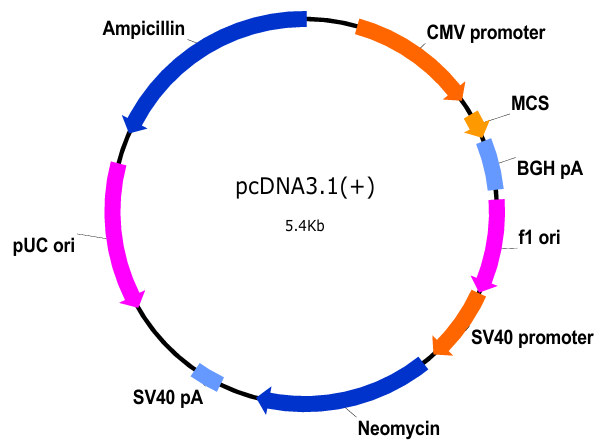

Supplement: Supplementary file 3 [file Data_sheet_3.docx]
