# Supplementary material for: Circular RNA circNUP214 serves as a microRNA-31 sponge to promote the progression of myasthenia gravis through NFAT5
Source: Front Neurol. 2026 Jul 9;17:1807844. doi: 10.3389/fneur.2026.1807844 (PMC13391849; doi:10.3389/fneur.2026.1807844)
Supplement: Supplementary file 4 [file Table_1.DOCX]

**Table S1** CircNUP214 expression in PBMCs stratified by clinical subtypes.

| Subgroup | n Median Range | *p* value |
| --- | --- | --- |
| All MG  **By MGFA**  Class I (Ocular)   \| Class II–IV (Generalized) \|  \| \| --- \| --- \|   **By antibody**  AChR positive  AChR + Titin ± RyR  Seronegative | 40 1.33 0.72 – 2.06  15 1.31 0.72 – 1.63  25 1.35 0.74 – 2.06  31 1.34 0.72 – 2.05  7 1.24 0.95 – 2.06  2 1.26, 1.60 (individual values) | 0.252  0.619 |

*p* values were calculated by Student's t-test (for normally distributed subgroups, Ocular vs Generalized) or Mann-Whitney U test (for AChR only vs Combined antibodies).
